# Enhanced *in vivo* thromboxane biosynthesis in melanoma compared with basal cell carcinoma skin cancers

**DOI:** 10.1093/skinhd/vzag120

**Published:** 2026-07-27

**Authors:** Giovanna Petrucci, Alessandro Di Stefani, Alessandro Rizzi, Lucia Di Nardo, Laura Del Regno, Mohammad A Farhoud, Ketty Peris, Bianca Rocca

**Affiliations:** Facoltà Dipartimentale di Medicina e Chirurgia, Università UniCamillus, Saint Camillus International University of Health Sciences, Rome, Italy; Dipartimento di Scienze Mediche e Chirurgiche Addominali ed Endocrino Metaboliche, UOC Dermatologia, Fondazione Policlinico Universitario A. Gemelli – IRCCS, Rome, Italy; Dipartimento di Medicina e Chirurgia Traslazionale, Sezione Dermatologia, Università Cattolica del Sacro Cuore, Rome, Italy; UOSD Diabetologia, Università Cattolica del Sacro Cuore, Rome, Italy; Dipartimento di Medicina e Chirurgia Traslazionale, Sezione Dermatologia, Università Cattolica del Sacro Cuore, Rome, Italy; Immunology Research Core Facility, Gemelli Science and Technology Park (GSTeP), Fondazione Policlinico Universitario A. Gemelli – IRCCS, Rome, Italy; UOSD Dermatologia, Ospedale ‘Andrea Tortora’, ASL Salerno, Pagani, Italy; Dipartimento di Medicina e Chirurgia Traslazionale, Sezione di Farmacologia, Università Cattolica del Sacro Cuore, Rome, Italy; Dipartimento di Scienze Mediche e Chirurgiche Addominali ed Endocrino Metaboliche, UOC Dermatologia, Fondazione Policlinico Universitario A. Gemelli – IRCCS, Rome, Italy; Dipartimento di Medicina e Chirurgia Traslazionale, Sezione Dermatologia, Università Cattolica del Sacro Cuore, Rome, Italy; Dipartimento di Medicina e Chirurgia, Libera Università Mediterranea (LUM), Casamassima (Bari), Italy

## Abstract

**Background:**

Activated platelets have been shown to promote tumorigenesis of several solid, largely gastrointestinal, tumours through the biosynthesis of cyclooxygenase-1-dependent thromboxane A_2_ (TXA_2_). The biosynthesis of the proinflammatory prostaglandin E_2_ (PGE_2_) has also been implicated in the progression of those solid tumours. Preclinical data suggest that platelet activation and PGE_2_ may contribute to melanoma progression, but clinical evidence is limited.

**Objectives:**

To investigate whether melanoma is associated with increased *in vivo* platelet activation and PGE_2_ biosynthesis, as assessed by urinary 11-dehydro-thromboxane B_2_ (11-dehydro-TXB_2_) and PGE_2_ metabolite (PGEM) levels, respectively, as compared with basal cell carcinoma (BCC) and healthy control samples; we also explored their potential relevance as biomarkers of melanoma progression.

**Methods:**

We measured urinary 11-dehydro-TXB_2_, a major TXA_2_ metabolite and a biomarker of *in vivo* platelet activation, as well as PGEM, in patients with melanoma and in patients with BCC, using age- and sex-matched healthy participants as controls. Thirty patients with stage 1–3 melanoma [mean (SD) age 57 (15) years; 21 men] and 24 patients with BCC [mean (SD) age 60 (12) years; 11 men] voluntarily participated in the study; 38 age- and sex-matched healthy individuals [mean (SD) age 56 (17) years; 26 men] from previously published studies by our group were included as controls.

**Results:**

Urinary 11-dehydro-TXB_2_ was higher in patients with melanoma [median 1284 pg mg^–1^ creatinine; interquartile range (IQR) 892–1784] vs. those with BCC (median 583 pg mg^–1^ creatinine; IQR 393–721) and healthy control participants (median 669 pg mg^–1^ creatinine; IQR 411–1184) (*P* < 0.001). Urinary 11-dehydro-TXB_2_ was comparable between patients with BCC and healthy control participants. We also studied six patients with stage 4 melanoma who showed the highest urinary 11-dehydro-TXB_2_ levels [1898.0 (IQR 1769.3–2622.1); *P* = 0.005 vs. all other groups]. PGEM levels did not differ among groups.

**Conclusions:**

Melanoma, unlike BCC, is associated with increased biosynthesis of biomarkers of activated platelets. Urinary 11-dehydro-TXB_2_ may be tested as a prognostic biomarker for melanoma progression. Moreover, whether inhibition of platelet activation may be an adjuvant chemopreventive strategy warrants further investigation.

## Graphical Abstract

**Graphical Abstract:**
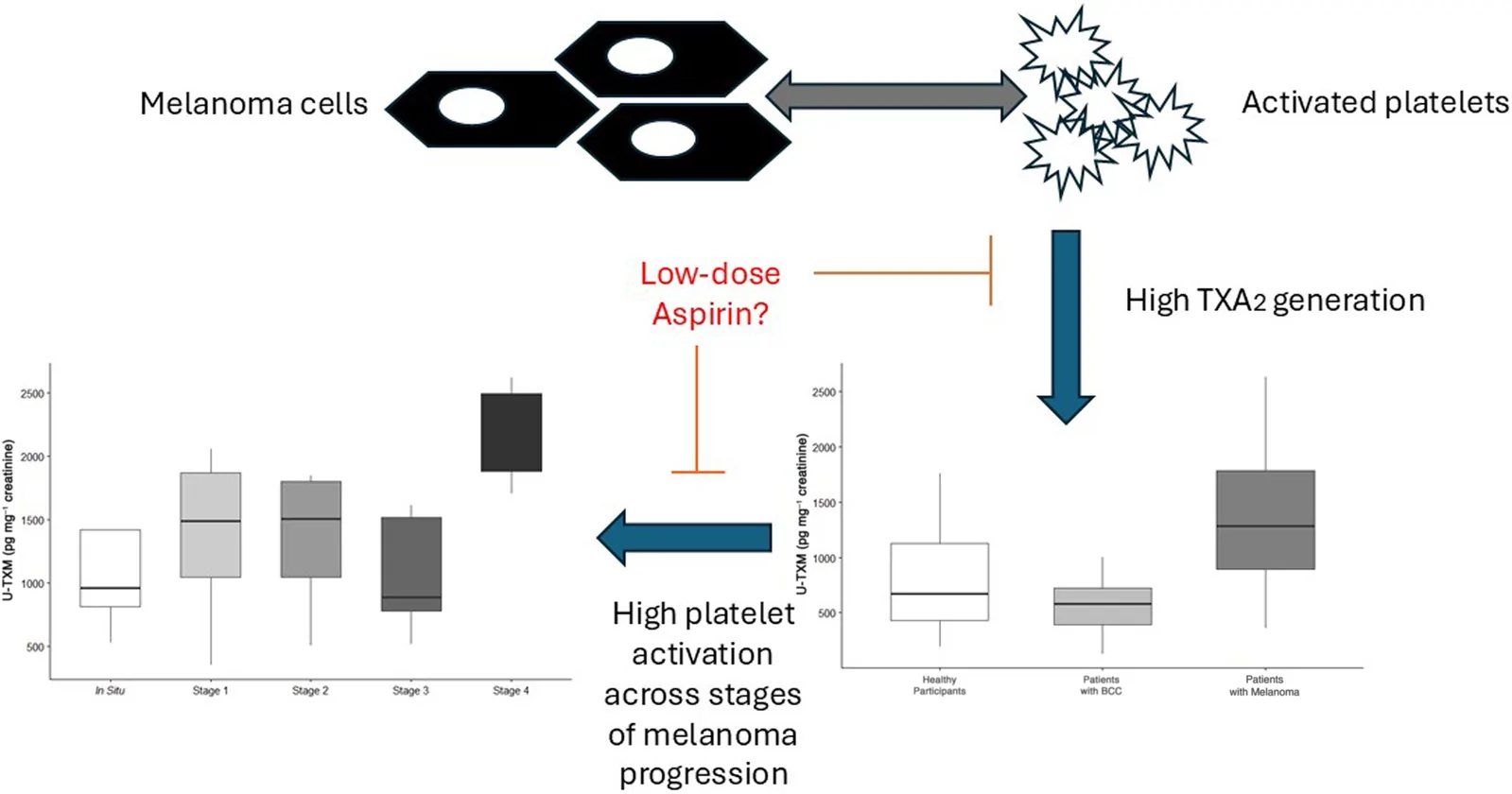

## Teaser

We investigated the *in vivo* biosynthesis of urinary 11-dehydro-thromboxane B_2_, a stable metabolite of thromboxane A_2_ largely derived from activated platelets, in patients with melanoma, compared with those with basal cell carcinoma skin cancers and healthy controls. We also measured *in vivo* biosynthesis of prostaglandin E_2_ through its urinary metabolite. Urinary 11-dehydro-thromboxane B_2_ levels were significantly higher in patients with melanoma, particularly in those with metastatic disease, whereas prostaglandin E_2_ metabolite levels did not differ among groups. The role of the thromboxane pathway and its inhibition in melanoma progression should be further explored.

What is already known about this topic?

- Platelet activation has been implicated in tumour growth, adhesion and metastasis, particularly in solid tumours of the gastrointestinal tract.
- Thromboxane A_2_, synthesized by platelet cyclooxygenase (COX)-1 activity, amplifies platelet activation, and its stable metabolite, 11-dehydro-thromboxane B_2_, is a validated biomarker of *in vivo* platelet activation.
- COX-2-mediated prostaglandin E_2_ synthesis promotes tumour proliferation, angiogenesis and immunosuppression in several solid cancers, including skin malignancies.

What does this study add?

- This study provides the first direct clinical evidence that melanoma, unlike basal cell carcinoma, is associated with enhanced *in vivo* thromboxane A_2_ biosynthesis, as measured by urinary 11-dehydro-thromboxane B_2_.
- Thromboxane A_2_ biosynthesis is particularly elevated in metastatic melanoma.
- These findings highlight thromboxane-dependent platelet pathways as potential prognostic biomarkers and therapeutic targets in melanoma, advancing the translational understanding of skin cancer biology.

Skin cancers comprise a heterogeneous group of neoplasms with distinct clinical, pathological and biological features. Among these, basal cell carcinoma (BCC) and squamous cell carcinoma are the most common, largely ultraviolet radiation-driven epithelial skin cancers. These tumours are typically indolent and curable by surgical excision, with survival rates approaching 100% in most cases.^1,2^ In contrast, melanoma is less prevalent but more aggressive, particularly in advanced stages. Age-adjusted melanoma incidence has increased by an average of 1.2% annually between 2013 and 2022, and in 2025 was estimated to account for ∼5% of all new cancer diagnoses.^3,4^ While the overall 5-year survival rate for melanoma is 94.7%, prognosis varies sharply by stage, approaching 100% for localized disease, around 70% with regional lymph node involvement and 35–40% for distant metastases.^5^

Over the past decade, accumulating evidence has linked platelet activation to multiple stages of tumorigenesis, including metastasis, mostly in solid cancer originating from the gastro­intestinal tract.^6–8^  *In vitro* studies show that activated platelets release or synthesize molecules that facilitate tumorigenesis, adhesion of circulating tumour cells to the endothelium and promote extravasation.^9,10^ Among platelet-derived bioactive mol­ecules, thromboxane A_2_ (TXA_2_), generated from arachidonic acid by the sequential action of cyclooxygenase (COX)-1 and thromboxane synthase, plays a key role in promoting and amplifying platelet activation. Having a short half-life, TXA_2_ is rapidly metabolized to thromboxane B_2_ (TXB_2_) and then enzymatically biotransformed into to stable, inactive metabolites excreted in urine, with 11-dehydro-TXB_2_ one of the most abundant, and serving as validated biomarker of *in vivo* platelet activation.^11^ In a large cohort from the Framingham Heart Study (*n* = 3044), high levels of urinary TXA_2_ metabolites at baseline were associated with increased cancer mortality [hazard ratio (HR) 1.63, 95% confidence interval (CI) 1.24–2.13; *P* = 0.0005], as well as cardiovascular (HR 2.41, 95% CI 1.71–3.39; *P* < 0.0001) and all-cause (HR 1.96, 95% CI 1.68–2.29; *P* < 0.0001) mortality over more than a decade of follow-up.^12^ Consistent with these findings, we have previously reported elevated urinary 11-dehydro-TXB_2_ levels in patients with colorectal, gastroesophageal, prostate and breast cancers.^13^

In line with the role of platelet activation in carcinogenesis, aspirin, a selective inhibitor of platelet COX-1-mediated TXA_2_ biosynthesis, has shown chemopreventive effects in several solid tumours, most convincingly in colorectal cancer, and in patients with Lynch syndrome.^14^ Moreover, several solid tumours, including skin cancers, express the COX-2 isoform, coupled with the downstream synthesis of prostaglandin E_2_ (PGE_2_).^15,16^ PGE_2_ ­promotes tumour growth through antiapoptotic, proangiogenic and immunosuppressive mechanisms mediated by distinct E-prostanoid receptor isoforms.^16,17^

While platelet activation and COX-2-mediated PGE_2_ generation have been extensively studied in gastrointestinal and other solid tumours, corresponding *in vivo* evidence in patients with skin cancers remains limited. Given the distinct biological and clinical characteristics of melanoma compared with BCC, we sought to determine whether platelet activation, as reflected by urinary 11-dehydro-TXB_2_, and PGE_2_ biosynthesis, as assessed by urinary PGE_2_ metabolites, differ between patients with melanoma and those with BCC.

## Materials and methods

### Study design and participants

We performed a cross-sectional study enrolling adult men and women (aged ≥18 years) with histologically confirmed nonmetastatic mela­noma or BCC who were observed at the Department of Dermatology, Catholic University, Rome, Italy. Melanoma cases included different stages of disease. Eligible participants were men and women with a confirmed diagnosis of melanoma or BCC. Exclusion criteria were smoking >5 cigarettes daily; severe hepatic, renal, cardiac or pulmonary disease; other malignant neoplasms within the past 5 years; type 2 diabetes mellitus; pregnancy or lactation; chronic use of nonsteroidal anti-inflammatory drugs (intake >3 days weekly); and current use of antiplatelet or anticoagulant agents.

The primary endpoint was the degree of *in vivo* platelet activation, as reflected by the noninvasive biomarker 11-dehydro­-thromboxane B_2_ (11-dehydro-TXB_2_), which is the major enzymatic metabolite of platelet-derived TXA_2_.^11^ 11-Dehydro-TXB_2_ levels were measured in patients with melanoma, BCC, and in age- and sex-matched healthy control individuals from previously published studies by our group^18,19^ or recruited ad hoc.

The secondary endpoint was the measurement of *in vivo* PGE_2_-dependent activity, assessed by a urinary PGE_2_ metabolite (PGEM).^20^ We also considered, in a secondary analysis, a subgroup of patients with metastatic melanoma.

### Laboratory measurements

Urine samples (5 mL) were collected at the time of clinical and dermoscopic diagnosis, that is, prior to or in the 2 weeks following skin tumour excision and, in all cases, before starting pharmacological therapy (if indicated) were aliquoted and stored at −80 °C until analysis. For 11-dehydro-TXB_2_, urine samples were processed as previously described.^13,21^ Briefly, 2 mL of urine aliquots was thawed at 37 °C and centrifuged at 340 ***g*** for 10 min; acidified to pH 3.5 with 60 µL 1 N acetic acid and spiked with 1500 cpm 3H-PGE_2_ (3.70–6.86 TBq mmol^–1^; Perkin Elmer, Boston, MA, USA) to calculate recovery. The urine samples were loaded into a 3 mL/200 mg C18 column (Bakerbond™-SPE; J.T. Baker, Gliwice, Poland) prewashed with 5 mL absolute ethanol, 5 mL bidistilled water and 5 mL 0.1 N acetic acid. The columns were then washed with 5 mL 15% ethanol and 5 mL petroleum ether, and eluted with 2 mL ethyl acetate; eluates were dried and reconstituted in 1 mL bidistilled water–acetonitrile–acetic acid (69.95:30:0.05 vol/vol/vol) buffer, and recovery was calculated by ^3^H-PGE_2_ counting. 11-Dehydro-TXB_2_ was measured with a standard enzyme-linked immunosorbent assay as previously described.^21,22^

PGEM was measured in urine samples using a commercial kit (item no. 514531; Cayman Chemical, Ann Arbor, MI, USA) following derivatization, according to the manufacturer’s instructions. This assay converts downstream metabolites of PGE_2_, such as 13,14-dihydro-15-keto PGE_2_ and 13,14-dihydro-15-keto PGA_2_, to stable derivatives that are quantified in immunoassays; the assay is suited to urine measurements with a sensitivity (80% B/B0) of 2 pg mL^–1^.^23^

Both urinary 11-dehydro-TXB_2_ and PGEM were expressed as pg per mg of urinary creatinine, measured by a commercial kit (Creatinine Colorimetric Detection Kit; Enzo Life Sciences, Farmingdale, NY, USA).

### Statistical analysis

Based on previous studies, we considered the median [interquartile range (IQR)] 11-dehydro-TXB_2_ excretion to be 434 (333–573) ng pg^–1^ creatinine based on 130 healthy participants from our database.^18,19,24^ As we hypothesized a ≥30% increase in 11-­dehydro-TXB_2_ excretion as biologically relevant, 30 individuals per group were needed with alpha = 0.05 and 80% power.

Continuous variables were expressed as mean (SD) or median (IQR), as appropriate. Group comparisons were performed using Anova for repeated measurements or paired *t*-tests. Correlations were assessed with Pearson or Spearman rank tests according to data distribution. A two-tailed *P*-value < 0.05 was considered statistically significant. Analyses were conducted with SigmaPlot 15 (Systat Software, San Jose, CA, USA).

## Results

A total of 30 patients with melanoma [mean (SD) age 57 (15) years; 21 men] and 24 patients with BCC [mean (SD) age 60 (12) years; 11 men] participated in the study. The main demographic and clinical characteristics of the patients are summarized in Table 1. Age- and sex-matched healthy individuals [*n* = 38, mean (SD) age 56 (17) years; 26 men; matching ratio 1:1 with the melanoma cohort] were also included as control participants.

**Table 1:** Baseline characteristics of patients with skin cancer included in this study

Characteristic	Melanoma (*n* = 30)	BCC (*n* = 24)
Age (years), mean (SD)	57.6 (15.5)	60 (12)
Male sex	21 (70)	11 (46)
Disease stage (*n*)	*In situ*, 4 (13); stage 1, 17 (57); stage 2, 4 (13); stage 3, 5 (17)	All stage 1
Comorbidities (*n*)^a^		
Yes	12	10
No	18	14
Comedications (*n*)	Antihypertensive drugs, 12	Antihypertensive drugs, 7
	Hypolipidaemic agents, 4; thyroid hormone replacement therapy, 1	Hypolipidaemic agents, 2

Data are presented as *n* (%) unless otherwise stated. *P*-value was nonsignificant for all different comparisons. BCC, basal cell carcinoma.

^a^Comorbidities were hypertension, benign prostatic hyperplasia, pulmonary fibrosis, depressive syndrome and hyperuricaemia.

Median urinary 11-dehydro-TXB_2_ levels were >2-fold higher in patients with melanoma compared with patients with BCC and healthy control participants, with median (IQR) values of 1284 (892–1784) pg mg^–1^ creatinine (*n* = 30), 583 (393–721) pg mg^–1^ creatinine (*n* = 24) and 669 (411–1184) pg mg^–1^ creatinine (*n* = 38), respectively (*P* < 0.001; Figure 1). No correlation was observed between 11-dehydro-TXB_2_ excretion and age, sex and the other collected variables listed in Table 1. The urinary 11-dehydro-TXB_2_ excretion levels measured in six patients with stage 4 melanoma were higher than all other groups (nonmetastatic melanoma, BCC and healthy individuals). Figure 2(a) shows the values of 11-dehydro-TXB_2_ excretion in the different stages of melanomas.

**Figure 1:**
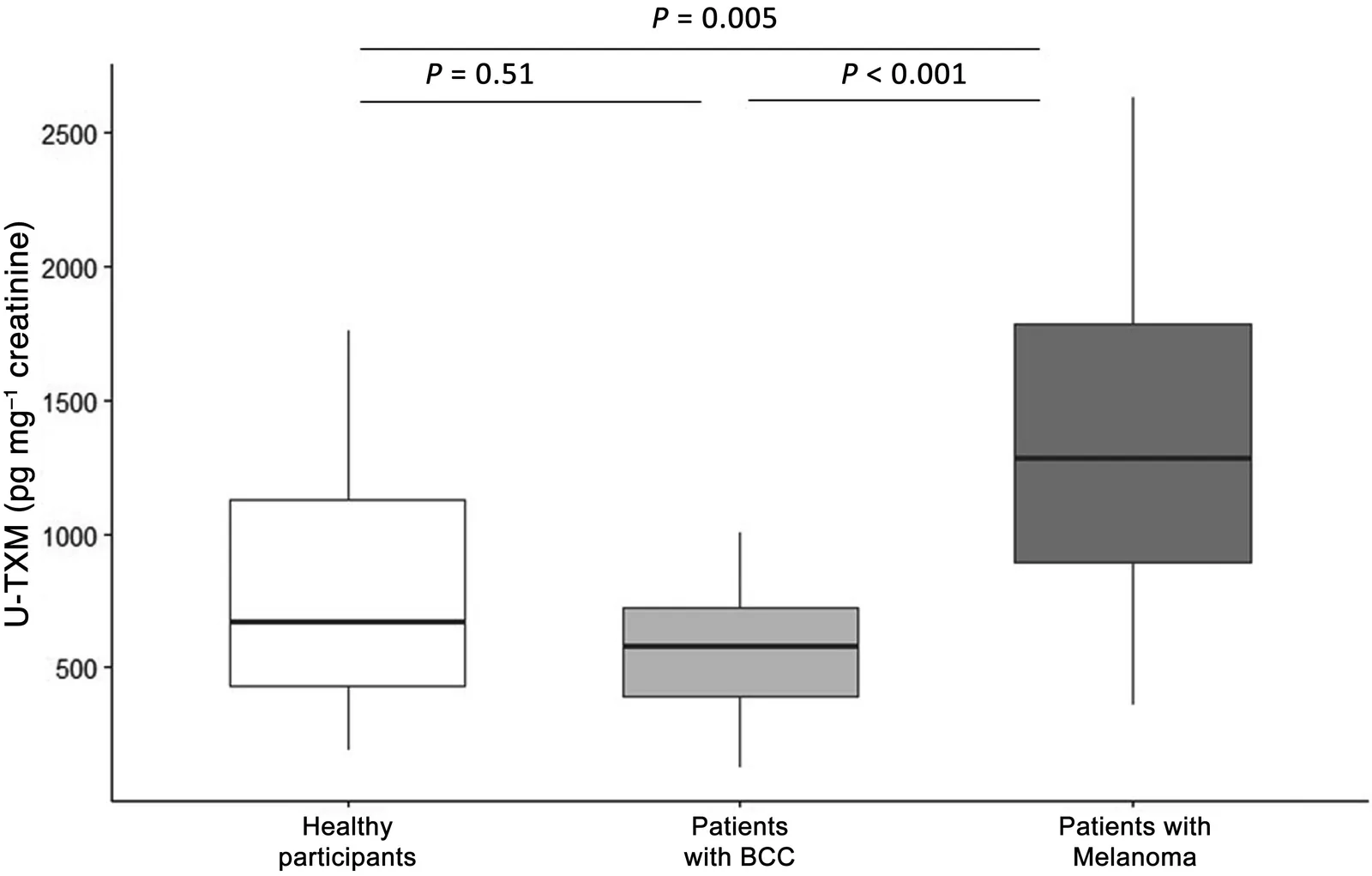
Urinary 11-dehydro-thromboxane B_2_ (U-TXM) levels in healthy participants, patients with melanoma and patients with basal cell carcinoma (BCC). Box plots represent the median and interquartile range. The 11-dehydro-thromboxane B_2_ levels were significantly higher in patients with melanoma compared with patients with BCC and healthy participants, while no significant difference was observed between healthy participants and patients with BCC.

**Figure 2:**
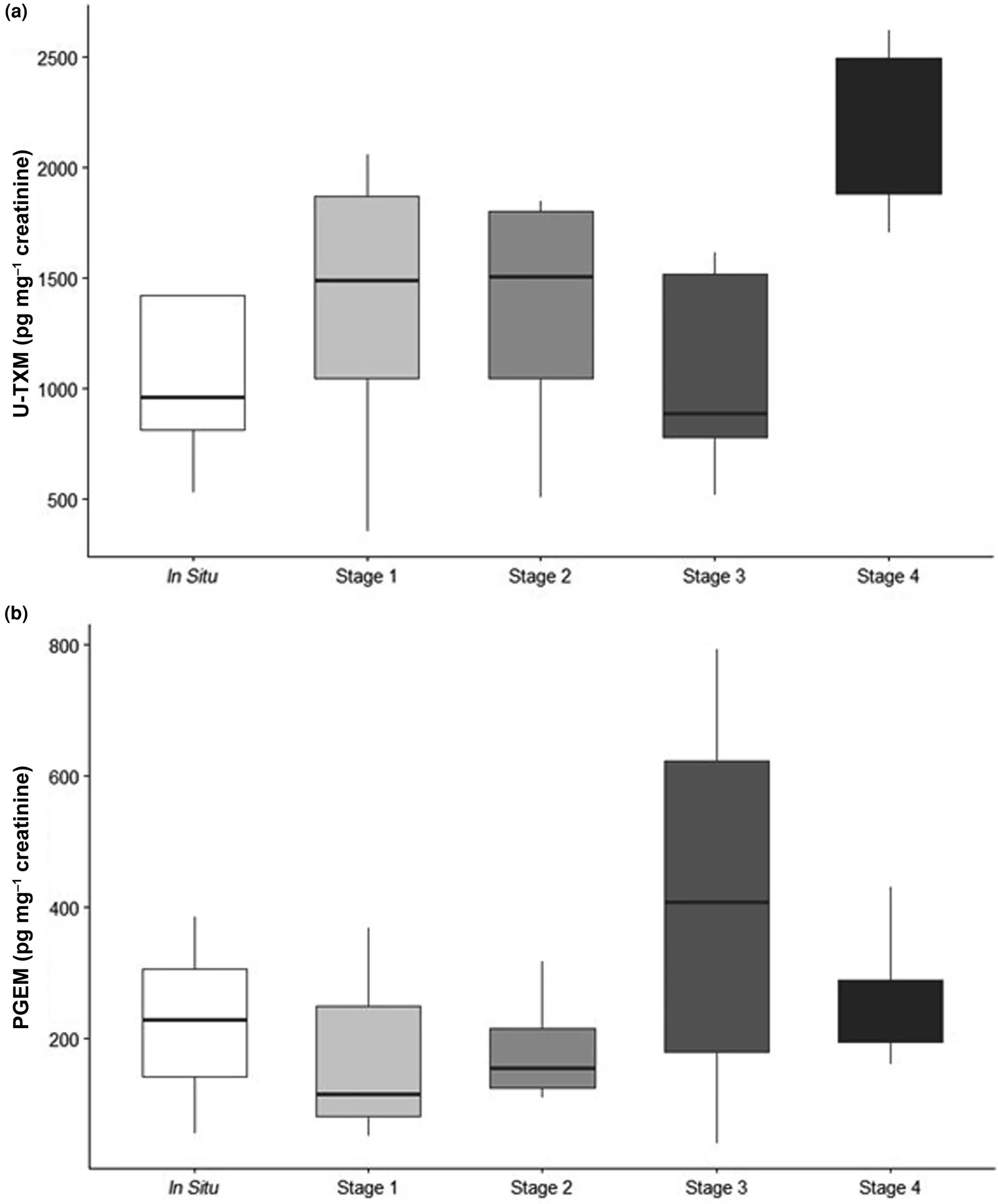
Urinary 11-dehydro-thromboxane B_2_ (U-TXM) and prostaglandin E_2_ (PGE_2_) metabolite (PGEM) levels in individuals with melanoma of different stages. Box plots represent the median and interquartile range. (a) The 11-dehydro-thromboxane B_2_ levels were significantly higher in stage 4 melanoma compared with all other melanoma stages. (b) Urinary PGEM levels were comparable among all melanoma stages.

In contrast to 11-dehydro-TXB2, urinary PGEM levels did not differ significantly among patients with melanoma (141 pg mg^–1^ creatinine, IQR 90–324), patients with BCC (120 pg mg^–1^ creatinine, IQR 89–209) or control groups (186 pg mg^–1^ creatinine, IQR 84–356) (*P* = 0.69). Urinary PGEM did not change across melanoma stage (Figure 2b).

PGEM levels were not correlated with 11-dehydro-TXB_2_, age, sex, hypertension or hypercholesterolemia.

## Discussion

In this study, we have shown, for the first time, that patients with cutaneous melanoma have significantly elevated urinary levels of urinary 11-dehydro-TXB_2_, a stable metabolite of TXA_2_, compared with patients with BCC and healthy control participants. With the limitations of a small subgroup, urinary 11-dehydro-TXB_2_ levels were particularly high in patients with metastatic melanoma.

TXA_2_ is biosynthesized predominantly (∼70%) by activated platelets and is a potent prothrombotic mediator, promoting platelet activation, aggregation and vasoconstriction. We previously reported increased 11-dehydro-TXB_2_ excretion in patients with gastrointestinal, breast and prostate cancers, and these high levels were substantially (∼60–70%) reduced by low-dose aspirin,^13^ a well-known platelet-selective COX-1 inhibitor.^25^ This pharmacological effect indicates that the excess in TXA_2_ generation observed in solid tumours originates from activated platelets, as in other cardiovascular conditions.^6^ The present study indicates that, among skin malignancies, enhanced platelet activation is associated with melanoma, particularly in the advanced phase.

Platelets are increasingly recognized to facilitate key steps in metastatization, including shielding circulating tumour cells from immune clearance, promoting endothelial adhesion and enabling extravasation.^26^ Specifically in melanoma, *in vitro* studies and preclinical models have shown that platelet-derived factors promote proliferation and metastatic progression.^27–31^ Robador *et al*. reported that von Willebrand Factor (vWF) derived from activated platelets can promote cerebral metastatic growth of melanoma in mice.^32^ Moreover, in preclinical studies, platelet inhibition by aspirin or clopidogrel reduced melanoma metastatic progression.^32–34^ Despite this compelling preclinical evidence, data on platelet activation and inhibition in human melanoma remain limited. High vWF levels, platelet–­leucocyte aggregates, soluble CD40L and CD62P were reported in the peripheral blood of patients with melanoma and brain metastases, and immunohistochemistry studies have shown vWF and platelet aggregate depositions in brain metastases.^32,35^ However, these biomarkers are not exclusively of platelet origin, as vWF is released by endothelial cells as well, and CD40L is produced by monocytes. By contrast, 11-dehydro-TXB_2_ derives largely from platelets, reflecting the degree of ongoing platelet activation,^11^ and it has been shown to predict cancer death in the large, prospective Framingham cohort, with more than 10 years of follow-up.^12^ In a longitudinal study with a 20-year follow-up, low-dose aspirin was associated with a 10% relative reduction of melanoma.^36^ Interestingly, a recent registry including 2419 patients with advanced, nonresectable melanoma showed a 30% increased progression-free survival associated with patients receiving low-dose aspirin on top of immune checkpoint inhibitors (HR 0.70, 95% CI 0.53–0.88; *P* = 0.004).^37^ As low-dose aspirin selectively inhibits platelet-derived TXA_2_, our findings provide mechanistic support to these associations. This study also suggests a role for antiplatelet therapy as an adjunctive strategy to the standard melanoma treatment. The significantly higher 11-dehydro-TXB_2_ levels in metastatic vs. nonmetastatic melanoma supports the hypothesis that platelet activation contributes to cancer progression. Clinically, our results may have two potential applications: platelet-derived biomarkers as tools for monitoring disease progression, and low-dose aspirin as adjunct chemopreventive treatment. Both warrant evaluation in larger prospective studies.

Different from melanoma, 11-dehydro-TXB_2_ levels in patients with BCC were lower and comparable to those of matched healthy participants, suggesting that enhanced platelet activation may be specific to aggressive skin tumours.

Unlike urinary 11-dehydro-TXB_2_, urinary PGEM, reflecting PGE_2_ biosynthesis *in vivo*, did not differ significantly among the melanoma, BCC and control groups. Considering that PGE_2_  *in vivo* is largely generated by COX-2-dependent mechanisms, this observation is apparently inconsistent with high COX-2 expression and PGE_2_ biosynthesis *in vitro* from isolated murine and human melanoma cells.^38^ However, local PGE_2_ autacoid release within tumour tissue may not necessarily alter systemic levels, and our study was not powered to detect small differences. Interestingly, a large observational study involving 16 142 cases of BCC and 631 of melanoma showed that COX-2 inhibitors administered to patients did not protect against skin cancer.^39^ Moreover, urinary PGEM was also unchanged in other cancers, such as ovarian cancer.^40^ Thus, the role of COX-2 inhibition in melanoma remains a topic of debate.^38^

This study has limitations. We did not test whether low-dose aspirin could reduce 11-dehydro-TXB_2_ levels in individuals with melanoma. While we have tested this intervention in other solid tumours associated with high 11-dehydro-TXB_2_ levels,^21^ proving the hypothesis of a platelet origin of the TXA_2_ generated *in vivo*, this was not investigated in the present study. Moreover, we did not assess longitudinal changes in 11-dehydro-TXB_2_ levels during disease progression or treatment. We did not measure systemic inflammatory markers (e.g. C-reactive protein and interleukin-6), which could have helped interpret the PGEM findings. In addition, other metabolites are generated from PGE_2_ biotransformation *in vivo*, in addition to those measured in the present study,^41^ but those metabolites have been previously validated.^23,39^ Finally, the group of patients with stage 4 disease was too small to provide strong data and we did not collect data during or after immunotherapy.

In conclusion, thromboxane biosynthesis, as reflected by urinary 11-dehydro-TXB_2_, is markedly elevated in melanoma, whereas no evidence of increased biosynthesis was found in patients with BCC. Our findings suggest that urinary 11-dehydro-TXB_2_ might be investigated in prospective cohorts as a biomarker of melanoma progression and provide a rationale for exploring whether antiplatelet strategies, such as low-dose aspirin, may be an adjuvant treatment added to standard chemotherapeutic agents.

## Data Availability

The data underlying this article will be shared on reasonable request to the corresponding author.
